# Clinical study of the effect of refractive status on stereopsis in children with intermittent exotropia

**DOI:** 10.1186/s12886-018-0822-2

**Published:** 2018-06-19

**Authors:** Dong Han, Danni Jiang, Jiahuan Zhang, Tianxu Pei, Qi Zhao

**Affiliations:** The Second Affiliated Hospital of Dalian Medical University, Dalian, China

**Keywords:** Intermittent exotropia, Stereopsis, Refractive status

## Abstract

**Background:**

Many studies have shown that patients with intermittent exotropia have different degrees of damage to their stereopsis function. The purpose of this study is to explore the effect of different refractive status on stereopsis in children with intermittent exotropia(IXT).

**Methods:**

We assessed 90 children of ages 4~ 16 years with intermittent exotropia at the Second Affiliated Hospital of Dalian Medical University during the years 2016–2017. According to their refractive status, the patients were divided into hyperopia group(spherical equivalent > or = − 1.00 diopters), myopia group(spherical equivalent < or = − 1.00 diopters), emmetropia group (spherical equivalent diopter: within ±1.00DS) and anisometropia group(binocular equivalent spherical difference > or = 1.50 diopter). The distant stereopsis of the patient was checked by the synoptophore, and the near stereopsis of the patients was checked by the Titmus stereogram. Then, we compared the difference between distant stereopsis and near stereopsis in the four groups.

**Results:**

(1)The retention rates of distant stereopsis in the hyperopia group, emmetropia group, myopia group and anisometropia group were 33.3, 45.2, 34.6, and 33.3%, respectively. There was no significant difference between the groups with different refractive status. (2)The retention rates of near stereopsis in the hyperopia group, emmetropia group, myopia group and anisometropia group were 66.7, 83.9, 80.8, and 55.6%, respectively. There were statistically significant differences between the emmetropia group and the anisometropia group (*P* = 0.030). (3)The retention rate of distant stereopsis in children with intermittent exotropia was 37.8%, and the retention rate of near stereopsis was 74.4%. The difference between the two groups was statistically significant (*P* = 0.019).

**Conclusions:**

The damage to distant stereopsis in children with intermittent exotropia is more serious than that to near stereopsis. The damage to near stereopsis in children with intermittent exotropia and anisometropiais more serious.

## Background

Intermittent exotropia (IXT) is a transitional strabismus between exophorias and constant exotropia, accounting for 50%~ 90% of exotropia [1]. It usually occurs before the age of 5. Women are more commonly affected than men, and Asians have a high incidence [2, 3]. Especially in China, the prevalence rate is estimated to be 3.9% [4]. Many studies have shown that patients with intermittent exotropia have different degrees of damage to their stereopsis function and that intermittent exotropia is more common with refractive error [5, 6]. Studies have shown that patients with intermittent exotropia usually have binocular foveal gaze and normal near stereopsis, [7] while other studies suggest that some patients with intermittent exotropia exhibit near stereopsis decline [8]. To investigate the effect of refractive error on the stereopsis in patients with intermittent exotropia, we summarized the clinical data of 90 patients with intermittent exotropia.

## Methods

### Subjects investigated

We assessed 90 children with intermittent exotropia in the pediatric ophthalmology clinic of the Second Affiliated Hospital of Dalian Medical University during the years 2016–2017. The study was approved by the Ethics Committee of the Second Affiliated Hospital of Dalian Medical University and it was performed in accordance with the tenets of the Declaration of Helsinki. A verbal consent was obtained from the parents of patients, and the oral consent has been approved by the Ethics Committee of the Second Affiliated Hospital of Dalian Medical University. Their mean age was (7.73 + 2.83) years, and they included 48 males and 42 females. Inclusion criteria were as follows: (1)the diagnosis of intermittent exotropia is clear. (2) Strabismus degree> or = 15. (3) Age of 4~ 16 years. (4) Ability to cooperate with astereopsis examination. (5) No monocular or binocular amblyopia and other ocular or systemic diseases.

### Division into groups

According to the refractive status, the subjects were divided into 4 groups: (1) the hyperopia group(spherical equivalent > or = − 1.00 diopters), (2) the myopia group(spherical equivalent < or = − 1.00 diopters), (3) the emmetropia group (spherical equivalent diopter: within ±1.00DS) and (4) the anisometropia group (binocular equivalent spherical difference > or = 1.50 diopter).

### Test methods

The clinical examination included the eye position, eye movement, visual acuity, prism cover test, cycloplegia, stereopsis examination, slit lamp examination, and fundus examination—all of which were analyzed by experienced ophthalmologists and optometrists.

1. The refractive status was examined by experienced optometrists after cycloplegia, with 1% atropine sulfate eye gel for subjects younger than 12 years and with compound tropicamide mydriatic eye drops for subjects older than 12 years.
2. We applied a synoptophore to examine for distant stereopsis. Binocular simultaneous vision was checked using printed pictures, one of a car and the other of a door; in this way, we could identify whether there was a coincident point. Then, we checked the fusion function using printed pictures with a butterfly and a cat, and we could determine whether there was a fusion function. We examined distant stereopsis using qualitative and quantitative analysis of stereoscopic pictures. The results were divided into existent distant stereopsis and nonexistent distant stereopsis. After that, we used a Titmus stereogram to examine near stereopsis, and the patients could identify the contents of the maximal arcsec picture recorded as existent near stereopsis, otherwise, the record was without near stereopsis.
3. We used the corneal reflection method and prism neutralization method to determine the nature of the strabismus and strabismus degree.

### Statistical method

We applied SPSS 20.0 statistical analysis software for the data analysis. The variance analysis was used to measure the age, strabismus degree and other measurement data among the four groups. Enumeration data of the number of patients with or without stereopsis was performed using the chi square test, and the independent sample t-test was used to measure the measurement data between the two groups.(*P* < 0.05 was considered statistically significant.)

## Results

There were 90 patients with intermittent exotropia, and their mean age was 7.73 + 2.83 years. The average near strabismus degree was (36.89 ± 8.06), and the average distant strabismus degree was (35.83 ± 13.62). The retention rate of distant stereopsis in the emmetropia group was 45.2%, while that of the children with refractive error was 33.9%; however, there was no significant difference between the two groups (*P* = 0.295). The retention rate of near stereopsis in the emmetropia group was 83.8%, while that of the children with refractive error was 69.5%; however, there was no significant difference between the two groups(*P* = 0.137). The mean age, deflection of eye position and average spherical equivalent of the intermittent exotropia patients with different refractive statuses are listed in Table 1.

**Table 1:** General conditions in patients with intermittent exotropia

Refractive status	Number	Age(years)	Right spherical equivalent(D)	Left spherical equivalent(D)	Near strabismus degree	Distant strabismus degree
Hyperopia	15	6.06 ± 1.53	2.45 ± 0.93	2.60 ± 0.98	37.33 ± 5.94	38.33 ± 6.45
Emmetropia	31	7.14 ± 2.98	0.01 ± 0.52	−0.02 ± 0.60	35.00 ± 6.32	35.97 ± 6.11
Myopia	26	8.63 ± 2.48	−2.19 ± 1.19	−2.24 ± 1.22	35.96 ± 8.13	30.77 ± 21.71
Anisometropia	18	8.84 ± 3.11	−1.82 ± 1.77	−2.38 ± 2.17	41.11 ± 10.79	40.83 ± 10.33
F		4.454	64.315	58.938	2.461	2.269
P		0.006	0.000	0.000	0.068	0.086
Total	90					

As shown in Table 2, The retention rates of distant stereopsis in children with intermittent exotropia was 37.8%, and the retention rate of near stereopsis was 74.4%. The difference between the two groups was statistically significant (*P* = 0.019). The retention rates of distant stereopsis in the hyperopia group, emmetropia group, myopia group and anisometropia group were 33.3, 45.2, 34.6, and 33.3%, respectively. The results revealed that the emmetropic group’s distant stereopsis retention rate was higher than that of the other three groups, but there was no significant difference among groups (*P* = 0.775). There was no significant difference between the groups with different refractive statuses. The retention rates of near stereopsis in the hyperopia group, emmetropia group, myopia group and anisometropia group were 66.7, 83.9, 80.8, and 55.6%, respectively. The retention rates of near stereopsis in the emmetropia group and myopia group were higher than those in the hyperopia group and anisometropia group. There was no significant difference between the anisometropia group and the myopia group (*P* = 0.071). but The difference between the anisometropia group and the emmetropia group was statistically significant (*P* = 0.030).

**Table 2:** Stereopsis in intermittent exotropia with different refractive states

		Emmetropia	Hyperopia	Myopia	Anisometropia	Total
Far stereopsis	yes	14(45.2%)	5 (33.3%)	9 (34.6%)	6 (33.3%)	34
	No	17(54.8%)	10(66.7%)	17(65.4%)	12(66.7%)	56
	Total	31	15	26	18	
near stereopsis	yes	26(83.9%)	10(66.7%)	21(80.8%)	10(55.6%)	67
	No	5 (16.1%)	5 (33.3%)	5 (19.2%)	8(44.4%)	23
	Total	31	15	26	18	90

## Discussion

Intermittent exotropia is the most common type of exotropia in the clinic. Among children with strabismus, the proportion with IXT has been reported to vary greatly by country, from 17% in the United States to 66% in Singapore, [9] and it accounts for approximately 25% of strabismus in children in the Western world [10, 11] and 44.9% in children in China [12]. It is a relatively higher burden in themainland of China [4, 13]. The position of the eyes can be normal, but strabismus becomes obvious with fatigue, illness, concentration or reading for a long time, and the patient often shuts his or her eyes when outdoors or in bright light. The patients’ eye position often changes between the orthoptic and oblique positions, retaining the opportunity for stereopsis development in the patients. Stereopsis of patients with intermittent exotropia alternates between development and loss, and thus, its near and far stereopsis can be damaged to varying degrees.

It is generally believed that the development of stereopsis begins at approximately 4 months after birth and gradually matures with age. Other study [14] added that foveal stereoacuity is acquired after the age of 6 years. At present, most scholars [15] believe that the near stereopsis of patients with intermittent exotropia is normal and that distant stereopsis is impaired. Lee [16] found that near stereopsis is associated with the age at diagnosis of intermittent exotropia and the best corrected vision, the older the age at diagnosis, the better the corrected vision, and the better the near stereopsis. In patients with intermittent exotropia, stereopsis is unsound with synoptophore fusion function damage appearing earlier and serious, internal and external fusional power dysplasia or internal fusional power dysplasia and external fusional power macroplasia. Besides, they only need image coherence sets and tension sets when they look at the distance, and strabismus is difficult to control. This also explains why children with intermittent exotropia exhibit the preservation of near stereopsis, while far stereopsis is more likely to be damaged.

We found that the patients of intermittent exotropia with myopia accounted for 28.9%, emmetropia accounted for 34.4%, hyperopia accounted for 16.7%, and anisometropia accounted for 20.0%. In children with intermittent exotropia, emmetropia and myopia are the most common, while hyperopia and anisometropia are less common. Ekdawi et al [17] conducted a retrospective study of 184 children under 19 years old who were diagnosed with IXT in the north central part of the United States, and found that the prevalence of myopia was over 90% in the 20 year old. Some studies have shown that intermittent exotropia with myopia and myopic astigmatism anisometropia are the main ones. There is a slight difference among the studies. This may be due to the difference in ages and sample sizes of the subjects, but the investigations report that intermittent exotropia with myopia is the main one. Because patients with myopia do not use or use less regulation in near fixation, the function of the converge is relatively weakened, leading to further reduction of the regulation and fusion function, and they are thus more prone to develop exotropia. For uncorrected myopia, because of its individual specific low AC / A, or without refractive correction, the near visual accommodation is impaired, which leads to the decrease of accommodative converge and leads to exotropia. And intermittent exotropia is prone to produce visual fatigue, which may have a certain effect on their stereopsis.

Min Yang MD et al [18] found that anisometropia accounted for 17.4% in 214 patients. Chia et al [19] reported 74 cases of anisometropia among 377 patients with concomitant exotropia (19.63%), which was consistent with our result that intermittent exotropia with anisometropia accounted for 20.0% of the cases. Kirwan and O’keefe[20] also believe that stereopsis is related to anisometropia, and that the greater the anisometropia, the greater the binocular visual function. Anisometropia has different degrees of effects on stereopsis; for binocular refraction, every 0.25 D difference can cause a 0.5% difference in the retinal image is 0.5%. The maximum difference of the retinal image is 5%, that is, the binocular diopter cannot be greater than 2.50 D. Beyond this threshold, the binocular images will be unequal in legibility and size, eventually resulting in fusion difficulties and decreased stereopsis. In addition, anisometropia can also cause the retinal images to vary in size, resulting in poor image fusion, high refractive power, and retinal suppression, which can cause exotropia. This study found that the retention rate of near stereopsis in the emmetropia group with intermittent exotropia was significantly higher than that in the anisometropia group (*P* = 0.030), which indicated that anisometropia had a great influence on near stereopsis. Moreover, greater anisometropia can easily lead to refractive amblyopia, and binocular vision must be greatly affected. Although this article did not analyze the children of intermittent exotropia with amblyopia, it is known that anisometropia has a great influence on stereopsis in children with intermittent exotropia, and we should pay attention to it.

Children with hyperopia have poor vision; both distant vision and near vision are markedly impaired. They are less stimulated by normal stimuli and are prone to form amblyopia, which can affect the development of stereopsis in children. We found that near stereopsis in patients with intermittent exotropia was more severe in their hyperopia group than in the emmetropia group and the myopia group, however, the difference was not statistically significant. Kim et al [21] reported that the near stereopsis of patients with intermittent exotropia and hyperopia is worse than that with emmetropia or myopia. Uncorrected hyperopia can get clear eye sight due to less regulation. The low activity collection mechanism results in low AC/A ratio and exotropia. And hyperopia often has the occurrence of amblyopia, which has a great influence on the development of stereovision in children with intermittent exotropia. Therefore, We should do further clinical studies with large samples.

In summary, as in many reports, [22, 23] the damage of distant stereopsis in children with intermittent exotropia is more severe than near stereopsis, and the refractive status has some influence on stereopsis in patients with intermittent exotropia, especially anisometropia, it has a greater effect on near stereopsis in children with intermittent exotropia. In addition, patients with refractive error are prone to develop intermittent exotropia. Intermittent exotropia not only affects the appearance but also increase the risk of refractive error in children and greatly affect the stereopsis of patients, which will have some psychological effects on children [24]. Consequently, we should pay attention to the refraction status of children with intermittent exotropia in order to correct the refractive error of these patients in a timely manner and stimulate the convergence function; if so, it will be easy to control the eye position, eventually enabling the children to have a better stereopsis.

## Conclusions

Our study demonstrated that the damage of distant stereopsis in children with intermittent exotropia is more severe than near stereopsis and anisometropia has a greater effect on near stereopsis in children with intermittent exotropia.

## Abbreviations

IXT: Intermittent exotropia
